# The Bed Nucleus of the Stria Terminalis–Deep Mesencephalic Nucleus Circuit Linking Emotion and Wakefulness

**DOI:** 10.1523/JNEUROSCI.1083-25.2025

**Published:** 2025-11-05

**Authors:** Zhongwen Zhang, Yoan Cherasse, Chandra Louis, Yuki C. Saito, Shingo Soya, Arisa Hirano, Takeshi Sakurai

**Affiliations:** ^1^Institute of Medicine, University of Tsukuba, Tsukuba 305-8575, Japan; ^2^Tsukuba Institute for Advanced Research (TIAR) and International Institute for Integrative Sleep Medicine (WPI-IIIS), University of Tsukuba, Tsukuba 305-8575, Japan; ^3^Doctoral Program in Biomedical Sciences, Graduate School of Comprehensive Human Sciences, University of Tsukuba, Tsukuba 305-8575, Japan; ^4^Life Science Center for Tsukuba Advanced Research Alliance (TARA), University of Tsukuba, Tsukuba 305-8575, Japan

**Keywords:** arousal, emotion, limbic system, sleep

## Abstract

The bed nucleus of the stria terminalis (BNST), a part of the extended amygdala, integrates emotional and arousal-related signals. While GABAergic BNST (GABA^BNST^) neurons have been implicated in promoting transitions from non-rapid eye movement (NREM) sleep to wakefulness, their downstream mechanisms remain unclear. Here, we identify a neuronal circuit through which GABA^BNST^ neurons promote arousal via projections to a midbrain region known as the deep mesencephalic nucleus (DpMe), located within the broader mesencephalic reticular formation. In male mice, we used a combination of optogenetics, fiber photometry, neural ablation, and tracing approaches to dissect this circuit. Optogenetic stimulation of GABA^BNST^ terminals in the DpMe during NREM sleep elicited rapid transitions to wakefulness and increased activity of glutamatergic DpMe (GLUT^DpMe^) neurons, as assessed by *c-fos* mRNA expression and calcium imaging. Similarly, an aversive air-puff activated GLUT^DpMe^ neurons, suggesting engagement by emotionally salient stimuli. Ablation of GLUT^DpMe^ neurons markedly attenuated arousal responses triggered by GABA^BNST^ stimulation, underscoring their essential role in this circuit. While monosynaptic rabies tracing revealed local input neurons to GLUT^DpMe^ cells, in situ hybridization identified few *Vgat-*positive interneurons among them. These findings suggest that GABA^BNST^ neurons may influence GLUT^DpMe^ neurons through noncanonical GABAergic mechanisms or via more complex local circuits beyond a simple disinhibition model. Together, these findings delineate a previously uncharacterized BNST–DpMe circuit that allows emotionally relevant stimuli to override sleep and promote arousal. This pathway may contribute to stress-related sleep disturbances and represents a potential target for therapeutic treatments for sleep disorders associated with emotional dysregulation.

## Significance Statement

We identified a neural circuit by which GABAergic neurons in the bed nucleus of the stria terminalis (GABA^BNST^ neurons) promote rapid transitions from non-rapid eye movement (NREM) sleep to wakefulness via projections to the deep mesencephalic nucleus (DpMe). Optogenetic stimulation of GABA^BNST^ neurons or exposure to aversive sensory stimuli activated glutamatergic DpMe (GLUT^DpMe^) neurons and triggered immediate arousal. Ablation of GLUT^DpMe^ neurons significantly attenuated this response, demonstrating their essential role. While monosynaptic tracing revealed local input neurons to GLUT^DpMe^ neurons, in situ hybridization detected few GABAergic interneurons among them, suggesting that this circuit involves more complex or noncanonical mechanisms beyond simple disinhibition. This BNST–DpMe pathway may underlie stress-related sleep disturbances and represent a promising target for therapeutic intervention.

## Introduction

The regulation of sleep and wakefulness has traditionally been attributed to two key processes: circadian rhythms and homeostatic sleep pressure (Borbély, 1982, 2016). However, growing evidence highlights the significant influence of emotional states on arousal regulation. Sleep and emotion are intricately intertwined through overlapping neural circuits and neuromodulatory systems, with dysregulation in one domain often disrupting the other. For instance, sleep disturbances such as insomnia are associated with increased emotional reactivity and a heightened risk for anxiety and mood disorders (Baglioni et al., 2010). Conversely, elevated emotional states—particularly anxiety—can disrupt sleep architecture by delaying sleep onset, reducing REM sleep, and increasing nocturnal awakenings (Goldstein and Walker, 2014).

A key brain structure involved in emotional processing and increasingly recognized for its role in arousal regulation is the bed nucleus of the stria terminalis (BNST). As part of the extended amygdala, the BNST integrates neuroendocrine, autonomic, and behavioral responses to emotionally salient stimuli and plays a pivotal role in stress adaptation (Crestani et al., 2013; Klumpers et al., 2017; Goode et al., 2019). The BNST is composed of both GABAergic and glutamatergic neurons (Daniel and Rainnie, 2015; Kim and Kim, 2021), receives strong noradrenergic input (Flavin and Winder, 2013), and exerts broad influence over anxiety, reward-seeking, and feeding behaviors (Kim et al., 2013). Its diverse connectivity enables the BNST to relay stress-related signals to downstream brain regions involved in arousal and motivation (Ortiz-Juza et al., 2021).

The functional versatility of the BNST arises from its complex microcircuitry and heterogeneous receptor expression. Notably, BNST hyperactivity has been linked to anxiety and stress-related disorders, highlighting its central role in emotional regulation (Goode et al., 2019). In addition to its role in emotion, the BNST has also been implicated in sleep–wake transitions. Prior studies demonstrated that optogenetic activation of GABA^BNST^ neurons during non-rapid eye movement (NREM) sleep rapidly induces arousal, suggesting their wake-promoting potential (Kodani et al., 2017). Consistent with this, Li et al. (2024) recently reported that GABA^BNST^ neurons are highly active during wakefulness and REM sleep, and their activation can promote arousal in part via projections to the ventral tegmental area (VTA; Li et al., 2024). This dual role in emotion and arousal suggests that the BNST may serve as a critical interface linking affective states to sleep–wake control.

Another key region involved in arousal is the deep mesencephalic nucleus (DpMe), a discrete nucleus located within the mesencephalic reticular formation (mRt). The DpMe contains predominantly glutamatergic neurons and has been implicated in sleep–wake regulation in previous physiological studies (Sakai, 2018). Its anatomical position within the broader mRt makes it well suited to integrate limbic and sensory signals that influence arousal state transitions. Anatomical studies have revealed dense projections from GABA^BNST^ neurons to the DpMe (Kodani et al., 2017), suggesting a functional circuit by which emotional inputs may influence arousal states.

In this study, we investigated the BNST–DpMe pathway and its role in sleep–wake regulation. We hypothesized that GABA^BNST^ neurons promote arousal by disinhibiting GLUT^DpMe^ neurons via local GABAergic interneurons within the DpMe. Using a combination of optogenetics, calcium imaging, viral tracing, and cell-type-specific ablation, we demonstrate that this circuit enables emotionally salient stimuli to override sleep and drive wakefulness. This pathway may underlie stress-induced sleep disruptions and represents a potential target for therapeutic intervention in hyperarousal-related sleep disorders.

## Materials and Methods

### Animals

All animal experiments were performed at the International Institute of Integrative Sleep Medicine (IIIS), University of Tsukuba, in accordance with its guidelines for animal experiments. The experimental protocols were approved by the Animal Experimentation Committee (Approval No. 24-065) and adhered to the guidelines of the US National Institutes of Health. C57BL/6J male mice (Charles River #000664), *Vgat-ires-Cre* (The Jackson Laboratory #016962), *Vglut2-ires-Cre* (The Jackson Laboratory #016963), and *Vgat-ires-FlpO* (The Jackson Laboratory #031331) male mice, 12–20 weeks of age and weighing 25–35 g, were used in this study. All mice were kept at 22°C with a 12 h light/dark cycle (9:00 A.M. = ZT0) and *ad libitum* access to food and water.

### Viral vectors

Adeno-associated virus (AAV) vectors were produced using a triple transfection, helper-free method in HEK293T cells. *SADΔG-GFP (EnvA)* was produced by transfecting B7GG cells with *pcDNA-SADB19L*, *pcDNA-SADB19G*, *pcDNA-SADB19N*, *pcDNA-SADB19P*, and *pSADΔG-GFP-F2*, followed by pseudotyping in BHK-RGCD-EnvA cells and subsequent purification by ultracentrifugation (Osakada and Callaway, 2013; Saito et al., 2018). The titers of recombinant AAV vectors were as follows: *AAV2-EF1a-DIO-ChR2-EYFP*, 9.88 × 10^11^ copies/ml; *AAV2-EF1a-DIO-GFP*, 4.06 × 10^12^ copies/ml; *AAV10-EF1a-fDIO-hChR2(H134R)-EYFP*, 7.57 × 10^13^ copies/ml; *AAV10-EF1a-fDIO-GFP*, 2.78 × 10^12^ copies/ml; *AAV10-CAG-DIO-RCaMP2*, 3.18 × 10^13^ copies/ml; *AAV10-EF1a-FLEX-taCasp3-TEVp*, 2.32 × 10^13^ copies/ml; *AAV10-CAG-nls-GFP*, 4.02 × 10^13^ copies/ml; *AAV10-EF1a-DIO-mCherry*, 1.61 × 10^13^ copies/ml; *AAV2-EF1a-FLEX-TVA-mCherry*, 7.98 × 10^13^ copies/ml; *AAV2-CAG-FLEX-RG*, 1.72 × 10^13^ copies/ml. Titers of *SADΔG-GFP(EnvA)* vectors were determined by infecting HEK293-TVA cell line and were found to be 1.2 × 10^9^ infectious units/ml.

### Surgery

Mice were deeply anesthetized using 1–2% isoflurane concentrations via a precision vaporizer and placed in a stereotaxic frame (David Kopf Instruments Model 942). A total of 120 nl of AAV vectors were stereotaxically injected into the BNST (coordinates: anterior-posterior: +0.14 mm from the bregma; mediolateral: ±0.7 mm; dorsoventral: −3.75 mm from surface of the brain) or into the DpMe (coordinates: anterior-posterior: −4.24 mm from the bregma; mediolateral: 1.0 mm; dorsoventral: −3 mm from surface of the brain). For optogenetic manipulations, optic fibers were implanted into the BNST (±10° angle to sagittal line, anterior-posterior: +0.14 mm from the bregma; mediolateral: ±1.5 mm; dorsoventral: −3.25 mm from surface of the brain) or into the DpMe (±10° angle to sagittal line, anterior-posterior: −4.24 mm from the bregma; mediolateral: ±1.75 mm; dorsoventral: −2.75 mm from surface of the brain). Two craniotomies were made at +1 mm lateral and +1.5 mm frontal to the bregma and lambda for EEG electrode implantation. EMG recordings were achieved by bilaterally inserting Teflon-coated silver wires into the neck muscles. The electrodes were secured with dental cement. After a recovery period of at least 2 weeks, the mice were transferred to the sleep recording chamber for further analysis.

### EEG/EMG recording

Mice were habituated to the recording conditions for 1 week, followed by two consecutive 24 h recording sessions. The average values from these two recording days were used as raw data, and data from all individual animals were included to determine their sleep/wakefulness patterns. EEG and EMG signals were amplified and filtered using an amplifier (BAS-8103P, Biotex; EEG, 0.5–250 Hz; EMG, 16–250 Hz), digitized at a sampling rate of 128 Hz, and recorded with EEG/EMG recording software (VitalRecorder, Kissei Comtec). Sleep stages were automatically scored and then manually corrected if necessary by an experienced researcher through visual inspection, classifying each 4 s epoch as wakefulness, NREM sleep, or REM sleep.

### Optogenetics

All optogenetic stimulation experiments were conducted during the light phase at ZT4–ZT8. Stimulation was manually triggered by the experimenter, with visual confirmation of behavioral state prior to each bout. Each mouse received stimulation at 5, 10, and 20 Hz in a counterbalanced order across days, with at least 24 h between stimulation sessions. Each session consisted of three stimulation bouts (20 s per bout). To minimize carry-over effects on sleep–wake states, no more than three bouts were delivered per day with an interbout interval of 1–2 h (at least 1 h).

#### Manipulation of GABA^BNST^ neurons or terminal

**Figure 1.**
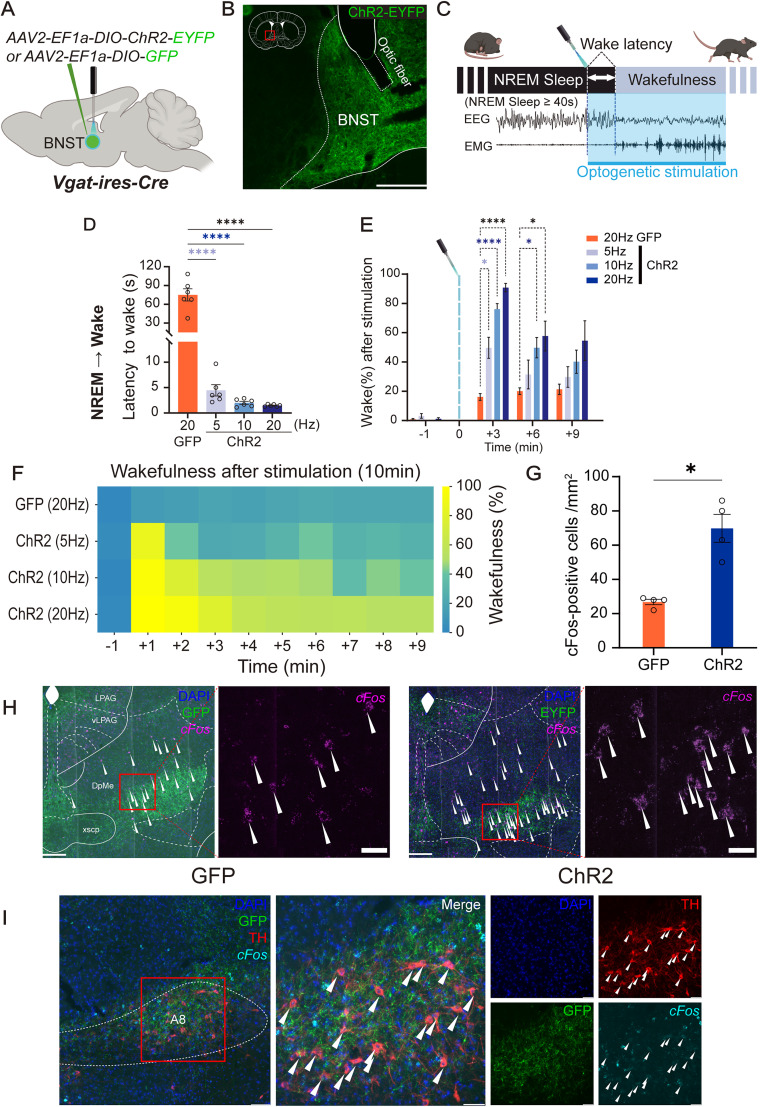
Optogenetic stimulation of the GABA^BNST^ neurons induced transitions from NREM sleep to wakefulness. ***A***, Schematic representation of GABA^BNST^ neurons optogenetic stimulation in the *Vgat-ires-Cre* mice. ***B***, Immunohistochemistry results illustrating ChR2-EYFP virus expression in the BNST, along with optic fiber localization in the BNST. Scale bar, 250 µm. ***C***, Illustration representing the protocol for laser stimulation during NREM sleep. GABA^BNST^ neurons in *Vgat-ires-Cre* mice were stimulated by blue laser during stable NREM sleep (≥40 s). A shaded blue box indicates optogenetic stimulation window, and blue line indicates timing of laser delivery. Stimulation parameters: wavelength, 462 nm; pulse frequency, 5/10/20 Hz; pulse width, 10 ms; duration, 20 s; laser power at fiber tip, 8–10 mW. ***D***, Quantification of the latency from NREM sleep to wakefulness induced by optogenetic stimulation of GABA^BNST^ neurons at various frequencies. Control (20 Hz), *n* = 7; ChR2 (5, 10 and 20 Hz), *n* = 7. One-way ANOVA revealed a significant effect of stimulation frequency (*F*_(3,20)_ = 54.62, *p* < 0.0001). Post hoc Dunnett's multiple-comparisons test showed that all ChR2 groups exhibited significantly shorter latencies compared with GFP controls (*p* < 0.0001 for all). ****p < 0.0001. ***E***, Wakefulness in 3 min time bins following optogenetic stimulation. All ChR2 groups (5, 10 and 20 Hz) exhibited significantly increased wakefulness compared with GFP controls in the first 3–6 min poststimulation. Two-way repeated-measures ANOVA revealed significant main effects of time (*F*_(1.978,39.55)_ = 85.93, *p* < 0.0001) and group (*F*_(3,20)_ = 14.76, *p* < 0.0001), as well as a significant interaction between time and group (*F*_(9,60)_ = 7.34, *p* < 0.0001). Post hoc Dunnett's multiple-comparisons test showed significantly higher values in the ChR2-20 Hz (*p* = 0.0162) and ChR2-10 Hz (*p* = 0.0143) groups compared with GFP, whereas ChR2-5 Hz was not significantly different (*p* = 0.532); *p < 0.05, **p < 0.01, ****p < 0.0001. ***F***, Heatmap showing the percentage of wakefulness during the 10 min following stimulation. The color scale represents wake percentage (yellow, high; blue, low). Each row represents a different group (GFP: 20 Hz; ChR2: 5, 10, and 20 Hz). ***G***, Number of *c-fos* mRNA-positive neurons in the DpMe after photostimulation. A significant increase was observed in ChR2-expressing mice (*n* = 4) compared with controls (*n* = 4), *p* = 0.0118; Welch's *t* test, *F*_(3,3)_ = 26.17, *p* = 0.0237. *p < 0.05. ***H***, Representative images of GFP- (left) or ChR2-EYFP-expressing (right) axon terminals in DpMe, costained with *c-fos* mRNA (magenta) and DAPI (blue). White arrows indicate *c-fos* mRNA-positive cells. Right images, Magnified views of boxed areas. Scale bars: overview, 250 µm; inset, 50 µm. ***I***, Representative images of ChR2-EYFP-expressing axon terminals in the A8 region, costained with tyrosine hydroxylase (red), *c-fos* mRNA (cyan), and DAPI (blue). White arrows indicate TH-positive cells. Right images, Magnified views of boxed areas. Scale bars: overview, 250 µm; inset, 50 µm.

**Figure 2.**
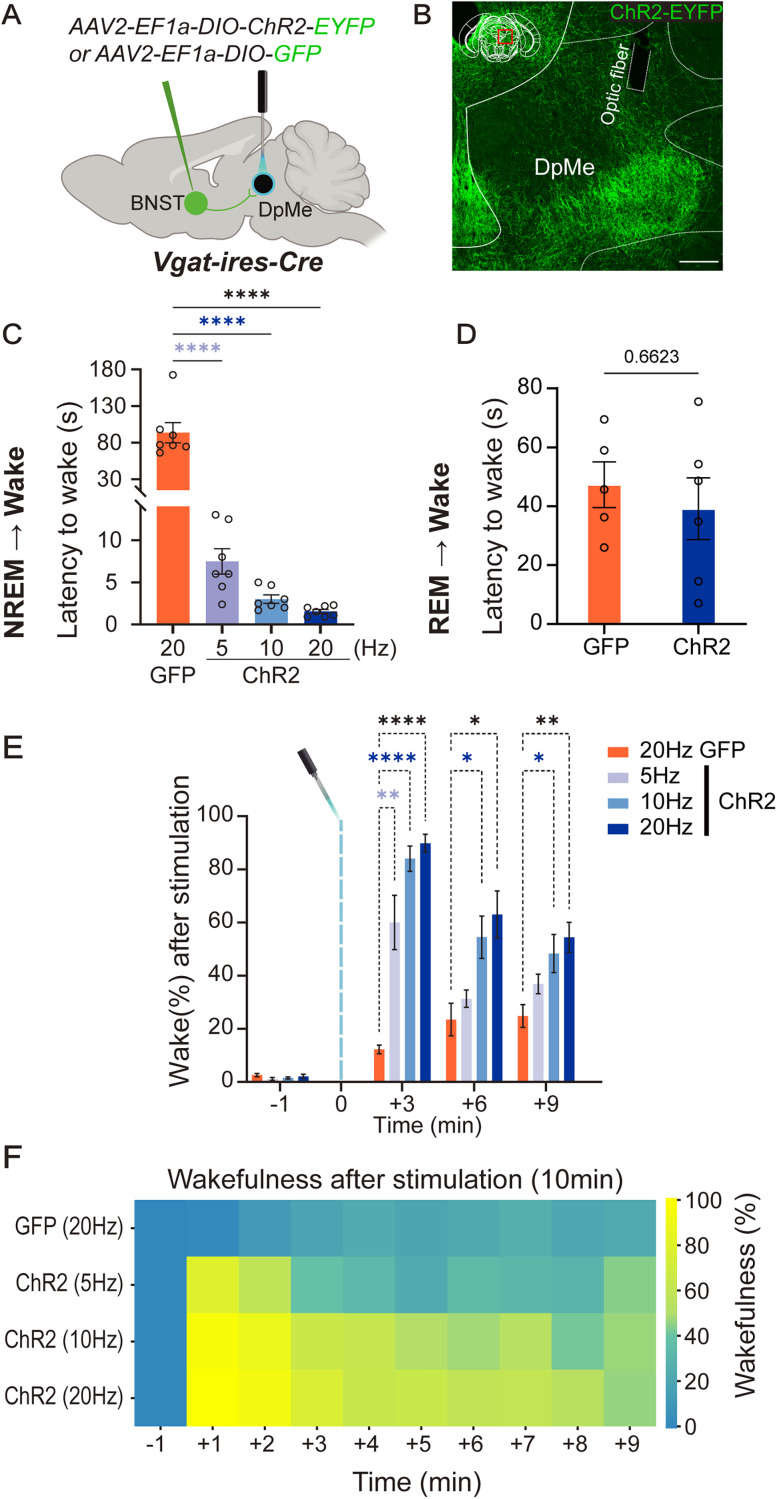
Optogenetic stimulation of the VGAT ^BNST → DpMe^ circuit induced transitions to wakefulness from NREM sleep but not from REM sleep. ***A***, Schematic representation of optogenetic stimulation of GABA^BNST^ neurons terminals in the DpMe in *Vgat-ires-Cre* mice. *AAV2-EF1a-DIO-ChR2-EYFP* or *AAV2-EF1a-DIO-GFP* was injected into the BNST, and optic fibers were implanted above the DpMe to manipulate BNST axons terminals. ***B***, Immunohistochemistry result showing ChR2-EYFP expression in GABA^BNST^-derived axons targeting the DpMe. The location of the optic fiber is indicated. Scale bar, 250 µm. ***C***, Quantification of latency from NREM sleep to wakefulness following optogenetic stimulation. ChR2-stimulated groups (5, 10, and 20 Hz) exhibited significantly shorter latencies compared with the GFP control (20 Hz) group. GFP, *n* = 7; ChR2 (5, 10, and 20 Hz), *n* = 7. Statistical analysis: one-way ANOVA revealed a significant effect of stimulation frequency (*F*_(3,24)_ = 41.99, *p* < 0.0001). Post hoc Dunnett's multiple-comparisons test showed that all ChR2 groups (5, 10, and 20 Hz) exhibited significantly shorter latencies compared with GFP controls (*p* < 0.0001 for all), ****p < 0.0001. The *y*-axis scale is expanded in this panel to accommodate the wider variability in wake latency observed in the GFP control group, which exhibited spontaneous arousals without external stimulation. This group naturally showed more dispersed and longer latencies compared with the optogenetically stimulated ChR2 groups. ***D***, Quantification of latency from REM sleep to wakefulness following 20 Hz optogenetic stimulation. No significant difference was observed between ChR2 (*n* = 6) and GFP (*n* = 5) groups. Mann–Whitney test; *p* = 0.6623. ***E***, Wakefulness percentages across 3 min time bins following optogenetic stimulation during NREM sleep. ChR2 groups (5, 10, and 20 Hz) showed significantly higher wakefulness than GFP controls within the first 6 min after stimulation. Two-way repeated-measures ANOVA revealed significant main effects of time (*F*_(2.361,56.65)_ = 113.0, *p* < 0.0001) and group (*F*_(3,24)_ = 24.03, *p* < 0.0001), as well as a significant interaction between time and group (*F*_(9,72)_ = 10.27, *p* < 0.0001); post hoc Dunnett's multiple-comparisons test showed significantly higher percentages in the ChR2-20 Hz (*p* < 0.0001), ChR2-10 Hz (*p* < 0.0001), and ChR2-5 Hz (*p* = 0.0081) groups compared with GFP controls. *p < 0.05, **p < 0.01, ****p < 0.0001. ***F***, Heatmap showing the percentage of wakefulness during the 10 min following stimulation. The color scale represents wake percentage (yellow, high; blue, low). Each row represents a different group (GFP: 20 Hz; ChR2: 5, 10, and 20 Hz).

To achieve specific expression of ChR2-EYFP in GABAergic neurons, we bilaterally injected *AAV2-EF1a-DIO-ChR2-EYFP* into the BNST of *Vgat-ires-Cre* mice. For the control group, we used an AAV expressing only GFP (*AAV2-EF1a-DIO-GFP*). Two optic fibers were implanted above the BNST or DpMe, and electrodes were implanted into the mice's heads to monitor EEG/EMG signals (Figs. 1*A*,*B*, 2*A*,*B*). After a 2 week recovery period, mice were transferred to a freely behaving sleep recording chamber. To allow habituation, the mice were connected to a cable-based sleep recording system at least 3 d before the experiment, enabling assessment of sleep–wake states via EEG/EMG signals. For the optogenetic stimulation, a laser (462 nm, 8 mW, 10 ms pulse width, 5, 10, or 20 Hz, 20 s duration; control group tested at 20 Hz) was activated after the mouse had maintained >40 s of NREM sleep (Fig. 1*C*).

#### Manipulation of GLUT^DpMe^ neurons

We bilaterally injected *AAV2-EF1a-DIO-ChR2-EYFP* into the DpMe of *Vglut2-ires-Cre* mice (*AAV2-EF1a-DIO-GFP* for the control group) and implanted optic fibers above the DpMe (Fig. 4*A*,*B*). After a 2 week recovery period, we followed the same optogenetics protocol as described above.

#### Fiber photometry of GLUT^DpMe^ neurons with the manipulation of GABA^BNST^ neurons

**Figure 3.**
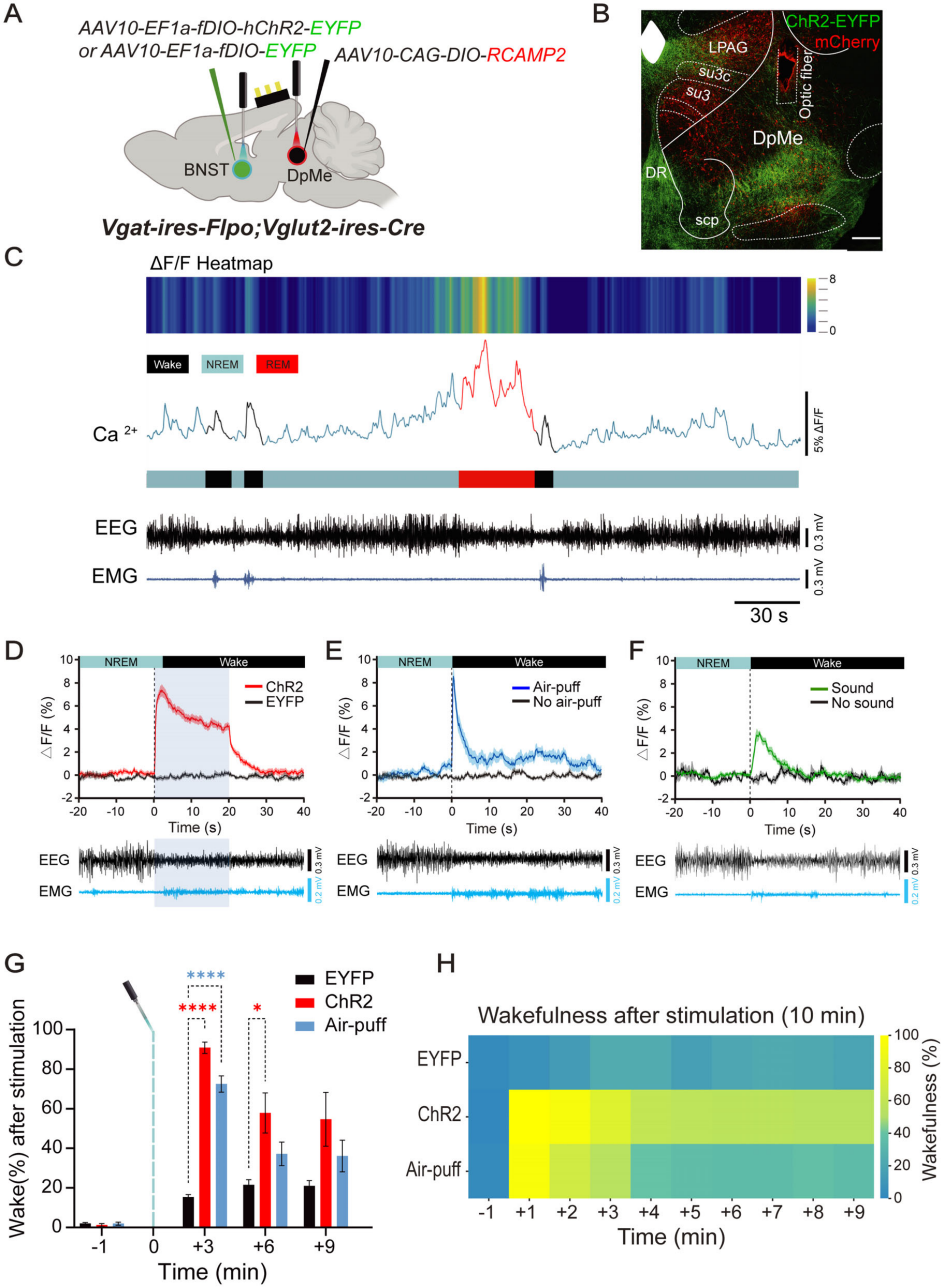
Glutamatergic DpMe neurons show rapid activation in response to BNST stimulation or aversive stimuli, and promote wakefulness. ***A***, Schematic of dual-virus fiber photometry experimental setup in *Vgat-ires-FlpO;Vglut2-ires-Cre* mice. *AAV2-EF1a-fDIO-ChR2-EYFP* or *AAV2-EF1a-fDIO-EYFP* was injected into the BNST for Flp-dependent expression in GABAergic neurons, and *AAV10-CAG-DIO-RCaMP2* was injected into the DpMe for calcium imaging in glutamatergic neurons. ***B***, Representative immunohistochemistry image confirming colocalization of GABA^BNST^ neuron projections and RCaMP2 expression in DpMe glutamatergic neurons. ***C***, Representative heatmap (top), Δ*F*/*F* calcium traces (middle), and corresponding EEG/EMG signals (bottom) during spontaneous sleep–wake transitions (light blue, NREM; red, REM; black, wake). ***D***, Group-averaged calcium responses (Δ*F*/*F*%) of glutamatergic DpMe neurons to optogenetic stimulation of GABA^BNST^ terminals (ChR2, *n* = 6). Shaded blue box indicates stimulation window (20 s, 20 Hz). ChR2 group shows robust calcium increase but not observed in controls (EYFP group). An unpaired, two-tailed Student's *t* test revealed a significant difference between groups (*t*_(10)_ = 10.55, *p* < 0.0001). Bottom panel shows EEG/EMG tracings. ***E***, Group-averaged calcium responses (Δ*F*/*F*%) of DpMe glutamatergic neurons to aversive stimulation (Air-puff, *n* = 6), which reliably induced transient activation compared with control (No air-puff group). An unpaired, two-tailed Student's *t* test revealed a significant difference between groups (*t*_(10)_ = 5.588, *p* = 0.0002). Bottom panel shows EEG/EMG tracings. ***F***, Group-averaged calcium responses (Δ*F*/*F*%) of DpMe glutamatergic neurons to auditory stimulation (Sound, *n* = 3), which reliably induced transient activation compared with control (No air-puff group). Welch's *t* test revealed a significant difference between groups (*t*_(3.439)_ = 8.710, *p* = 0.0018). Bottom panel shows EEG/EMG tracings. ***G***, Wakefulness percentages after stimulation (binned in 3 min intervals). Both ChR2- and air-puff-stimulated animals showed significantly higher wakefulness than EYFP group in the early poststimulation period. Two-way repeated-measures ANOVA revealed significant main effects of time (*F*_(1.405,21.07)_ = 72.51, *p* < 0.0001) and group (*F*_(2,15)_ = 18.59, *p* < 0.0001), as well as a significant interaction between time and group (*F*_(6,45)_ = 11.72, *p* < 0.0001). Post hoc Dunnett's multiple-comparisons test showed that, at +3 min, both ChR2 (*p* < 0.0001) and Air-puff (*p* < 0.0001) groups exhibited significantly higher percentages compared with EYFP controls, and at +6 min, ChR2 remained significantly elevated (*p* = 0.0252). *p < 0.05, ****p < 0.0001. ***H***, Heatmap of wakefulness percentages during the 10-minutes period following stimulation. Each row denotes one group (EYFP, ChR2, Air-puff).

We utilized *Vgat-ires-FlpO;Vglut2-ires-Cre* mice, which express flippase recombinase in GABAergic neurons and Cre recombinase in glutamatergic neurons. We bilaterally injected *AAV10-EF1a-fDIO-hChR2-EYFP* into the BNST to enable optogenetic stimulation of GABA^BNST^ neurons, while *AAV10-CAG-DIO-RCaMP2* was injected into the DpMe to allow real-time calcium imaging of GLUT^DpMe^ neuronal activity in *Vgat-ires-Cre* mice. Optic fibers were implanted above the BNST and DpMe for targeted stimulation and fiber photometry recording, respectively (Fig. 3*A*,*B*). Following a recovery period and habituation, mice underwent EEG/EMG recordings to classify sleep–wake states.

#### Ablation of GLUT^DpMe^ neurons with optogenetic excitation of GABA^BNST^ terminals in the DpMe

ChR2 was selectively expressed in GABA^BNST^ neurons through injection of a Flp-dependent AAV carrying ChR2 (*AAV10-EF1a-fDIO-hChR2-EYFP*) into the BNST, followed by bilateral implantation of optic fibers above the DpMe in *Vgat-ires-FlpO;Vglut2-ires-Cre* mice. To ablate GLUT^DpMe^ neurons, we injected Cre-dependent AAV carrying Caspase-3 into the DpMe. To verify successful ablation, two additional AAVs were coinjected: *AAV10-CAG-nls-GFP* for confirming injection site accuracy and *AAV10-EF1a-DIO-mCherry* to confirm glutamatergic neurons death. In the control group, *AAV10-EF1a-FLEX-taCaspase3-TEVp* injection was omitted (Fig. 5*A*,*B*).

### Immunohistochemistry

Animals were deeply anesthetized with isoflurane, followed by perfusion with phosphate buffered saline (PBS), and subsequently with 4% paraformaldehyde in PBS (4% PFA). Brains were then extracted and postfixed in 4% PFA at 4°C overnight, followed by transfer to 30% sucrose in PBS at 4°C. After overnight incubation, brains were embedded in Tissue-Tek O.C.T. Compound (Sakura Finetek Japan; catalog #4583) and stored at −80°C. Coronal brain sections were cut at a thickness of 30 µm using a cryostat (Leica CM1860 UV).

Serial brain sections were rinsed three times with PBS and blocked with PBS containing 0.25% Triton X-100 plus 3% BSA (blocking solution) for 1 h. The sections were then incubated with primary antibodies diluted in blocking solution at 4°C overnight. Following this, sections were rinsed six times with PBS and incubated with secondary antibodies diluted in blocking solution at 4°C overnight. The sections were counterstained with Nissl stain (1:500; Thermo Fisher Scientific, catalog #N21482, RRID: AB_2620170) or 4′,6-diamidino-2-phenylindole (DAPI; Thermo Fisher Scientific, catalog #D3571, RRID: AB_230744). After counterstaining, the sections were rinsed with PBS, mounted, and coverslipped. The primary antibodies used in this study were as follows: rat anti-GFP (1:1,000, monoclonal, GF090R, Nacalai Tesque) and goat anti-mCherry antibody (1:1,000, catalog #AB0081-200, SICGEN). Images were obtained with a confocal microscope (Leica TCS SP8 STED 3X).

### Fluorescence in situ hybridization

To detect *Vgat*, *Vglut2*, and *c-fos* mRNA, fluorescence in situ hybridization (FISH) was performed according to the manufacturer's protocol (RNAscope Fluorescent Multiplex Reagent Kit, catalog #320850, Advanced Cell Diagnostics). After fixation and cryoprotection, brains were embedded in Tissue-Tek O.C.T. Compound (Sakura Finetek; catalog #4583) and stored at −80°C. Coronal brain sections were then cut at thickness of 20 µm using a cryostat (Leica CM1860 UV). The following probes were used for hybridization: Mm-Slc32a1 (#319191); Mm-Slc17a6 (#319171); Mm-Fos-C2 (#316921-C2); Mm-Slc32a1-C3 (#319191-C3). Fluorescence images were acquired using a confocal microscope TCS SP8 STED 3X (Leica Biosystems). For quantification of *c-fos* mRNA-positive neurons, manual counting was performed by an experimenter blinded to the experimental conditions. Anatomical boundaries were identified using DAPI counterstaining and the Allen Mouse Brain Atlas as a reference. No automated software was used for quantification.

### Retrograde tracing

To identify neurons that make direct synaptic contact with GLUT^DpMe^ or GABA^DpMe^ neurons, we injected *AAV2-EF1a-FLEX-TVA-mCherry* and *AAV2-CAG-FLEX-RG* into the DpMe of *Vglut2-ires-Cre* mice or *Vgat-ires-Cre* mice, respectively. Two weeks later, *SADΔG-GFP(EnvA)* was injected into the DpMe (Fig. 6*A*). One week postinjection, immunohistochemistry revealed the GLUT^DpMe^ or GABA^BNST^ neurons expressing both mCherry and GFP signals (starter cells), while input neurons only displayed GFP signals.

### Statistics

Statistical analyses were conducted using Prism version 10 software (GraphPad). In optogenetic experiments, one-way ANOVA test was used to analyze the difference in the transition latency from NREM sleep to wakefulness between the ChR2 groups (5, 10, 20 Hz) and the control group (20 Hz). The percentage of wakefulness after optogenetic stimulation was analyzed by two-way repeated-measures ANOVA to detect significant effects of time, followed by Dunnett's multiple-comparisons test for every 3 min analysis.

## Results

### Excitation of GABA^BNST^ neurons promotes wakefulness

To confirm the role of GABAergic neurons in the BNST (GABA^BNST^ neurons) in modulating sleep–wake transitions, we first examined the effect of their optogenetic activation during NREM sleep. Channelrhodopsin-2 (ChR2) was expressed in GABA^BNST^ neurons, and optic fibers were implanted into the BNST. Stimulation was applied at varying frequencies (5, 10, and 20 Hz; Fig. 1*A–C*). Compared with GFP-expressing control mice, all ChR2-expressing groups exhibited a rapid transition from NREM sleep to wakefulness following stimulation (Fig. 1*D*; GFP-20 Hz, *n* = 6, 74.94 ± 9.72 s; ChR2-20 Hz, *n* = 6,1.47 ± 0.09 s; ChR2-10 Hz, *n* = 6, 2.01 ± 0.3 s; ChR2-5 Hz, *n* = 6, 4.45 ± 1.12 s; one-way ANOVA test, *F*_(3,20)_ = 54.62, *p* < 0.0001). Regarding the choice of stimulation frequencies, we selected 5, 10, and 20 Hz to span a physiologically plausible range of firing rates for BNST^GABA^ neurons. Although the precise in vivo activity of these neurons under different behavioral states remains incompletely characterized, similar frequencies have been used in prior studies to evoke robust behavioral responses through BNST GABAergic pathways (Marcinkiewcz et al., 2016). These precedent studies support the validity and relevance of our stimulation parameters for investigating BNST-driven arousal circuits.

Wakefulness was sustained following stimulation, with all ChR2 groups spending significantly more time awake over a 10 min poststimulation period compared with controls (Fig. 1*E*,*F*). During the first 3 min after stimulation, wakefulness was markedly increased in all ChR2 groups, with higher frequencies producing stronger effects (GFP-20 Hz, 15.8 ± 2.4%; ChR2-20 Hz, 90.67 ± 2.82%, *p* < 0.0001; ChR2-10 Hz, 76.02 ± 3.82%, *p* <0.0001; ChR2-5 Hz, 49.67 ± 7.17%; *p* = 0.0099). Although the wake-promoting effect declined over time, it remained significantly elevated at 6 min poststimulation in the 20 Hz and 10 Hz groups (GFP-20 Hz, 19.3  2.14%; ChR2-20 Hz, 66.13 ± 10.78%, *p* = 0.0162; ChR2-10 Hz, 49.75 ± 6.95%, *p* = 0.0143; ChR2-5 Hz, 31.45 ± 9.84%, *p* = 0.532; two-way repeated-measures ANOVA; time, *F*_(1.978,39.55)_ = 85.93, *p* < 0.0001; group, *F*_(3,20)_ = 14.76, *p* < 0.0001; interaction, *F*_(9,60)_ = 7.34, *p* < 0.0001; post hoc comparisons, Dunnett's multiple-comparisons test).

Additionally, we observed dense ChR2-EYFP-positive axonal projections from GABA^BNST^ neurons to the DpMe. This was accompanied by a significant increase in *c-fos* mRNA expression in neurons in the DpMe 30 min after GABA^BNST^ neurons stimulation (Fig. 1*G*,*H*; GFP-20 Hz, *n* = 4, 26.75 ± 3.2 cells/mm^2^, ChR2-20 Hz, *n* = 4, 69.75 ± 16.38 cells/mm^2^, *p* = 0.0118; Welch's *t* test, *F*_(3,3)_ = 26.17, *p* = 0.0237).

Previous work has shown that BNST GABAergic neurons can promote arousal via dopaminergic pathways, including projections to midbrain dopamine neurons (Li et al., 2024). To test whether such a mechanism might be involved in our BNST → DpMe circuit, we examined the potential engagement of retrorubral dopaminergic neurons in the A8 region. Specifically, we conducted dual-labeling in situ hybridization for *c-fos* mRNA and immunohistochemistry for Tyrosine Hydroxylase (TH) in the DpMe region 30 min after optogenetic stimulation of GABA^BNST^ neurons during NREM sleep. Notably, TH-positive neurons showed no *c-fos* mRNA expression, while robust *c-fos* mRNA labeling was observed in nearby TH-negative neurons (Fig. 1*I*). These findings indicate that the arousal-promoting effects observed in our study are unlikely to involve activation of retrorubral dopaminergic neurons and instead suggest that nondopaminergic populations within the DpMe mediate this effect.

### Activation of GABA^BNST^ fibers in DpMe induces rapid transition from NREM sleep to wakefulness

To further examine the contribution of the *Vgat* ^BNST → DpMe^ pathway in wake promotion, we examined the effects of optogenetic activation of GABA^BNST^ axon terminals within the DpMe during NREM sleep (Fig. 2*A*,*B*). Light stimulation was applied after mice had sustained at least 40 s of NREM sleep. Terminal stimulation significantly shortened the latency to wakefulness in ChR2-expressing mice compared with GFP controls (Fig. 2*C*; GFP-20 Hz, *n* = 7, 93.54 ± 13.75 s; ChR2-20 Hz, *n* = 7, 1.56 ± 0.23 s, *p* < 0.0001; ChR2-10 Hz, *n* = 7, 3.01 ± 0.50 s, *p* < 0.0001; ChR2-5 Hz, *n* = 7, 7.48 ± 1.51 s, *p* < 0.0001; one-way ANOVA, *F*_(3,24)_ = 41.99, *p* < 0.0001; post hoc comparisons, Dunnett's multiple-comparisons test). In contrast, stimulation of GABA^BNST^ terminals in the DpMe during REM sleep failed to induce rapid transitions to wakefulness in both ChR2 and control groups (Fig. 2*D*; GFP-20 Hz, *n* = 5, 47.3 ± 7.77 s; ChR2-20 Hz, *n* = 6, 39.13 ± 10.48 s, *p* = 0.6623, Mann–Whitney test), indicating state-dependent responsiveness of this pathway.

ChR2-expressing mice also exhibited significantly longer periods of wakefulness after stimulation compared with controls (Fig. 2*E*,*F*). During the first 3 min poststimulation, wakefulness was markedly increased in all ChR2 groups relative to the GFP group, with higher stimulation frequencies inducing stronger responses (GFP-20 Hz, 12.24 ± 1.6%; ChR2-20 Hz, 89.81 ± 3.35%, *p* < 0.0001; ChR2-10 Hz, 84.06 ± 4.8%, *p* < 0.0001; ChR2-5 Hz, 60.01 ± 10.26%; *p* = 0.0081; two-way repeated-measures ANOVA; time, *F*_(2.361,56.65)_ = 113.0, *p* < 0.0001; group, *F*_(3,24)_ = 24.03, *p* < 0.0001; interaction, *F*_(9,72)_ = 10.27, *p* < 0.0001; post hoc comparisons, Dunnett's multiple-comparisons test). While arousal levels gradually declined over time, they remained significantly elevated at later time points in the 10 and 20 Hz conditions, indicating sustained wakefulness following stimulation.

### Activation of GABA^BNST^ neurons triggers transient excitation of GLUT^DpMe^ neurons

Previous studies have implicated the deep mesencephalic nucleus (DpMe) in arousal regulation, and in particular, Sakai (2018) reported that a population of glutamatergic neurons in the DpMe (GLUT^DpMe^ neurons) exhibits state-dependent activity patterns associated with wakefulness (Sakai, 2018). Based on these findings, we hypothesized that these neurons might serve as key downstream effectors of GABA^BNST^ input. To investigate how activation of GABA^BNST^ neurons affects the activity of GLUT^DpMe^ neurons, we combined fiber photometry, optogenetics, and sleep state monitoring in *Vgat-ires-FlpO;Vglut2-ires-Cre* mice. ChR2 was selectively expressed in GABA^BNST^ neurons, and RCaMP2 was expressed in GLUT^DpMe^ neurons. EYFP was expressed in GABA^BNST^ neurons instead of ChR2 in control mice (Fig. 3*A*,*B*).

Calcium signal dynamics (Δ*F*/*F*) revealed distinct patterns of GLUT^DpMe^ neuron activity across sleep states. A representative heatmap and trace showed elevated Ca^2+^ activity during REM sleep (red), which sharply decreased just prior to awakening (black), while activity remained low and stable during NREM sleep (Fig. 3*C*, light blue).

Optogenetic stimulation of GABA^BNST^ neurons during NREM sleep (20 Hz) evoked a robust, transient increase in GLUT^DpMe^ calcium activity that coincided with NREM-to-wake transitions (Fig. 3*D*; EYFP, *n* = 6, 0.16 ± 0.25%; ChR2, *n* = 6, 7.28 ± 0.85%, *p* < 0.0001; two-tailed, unpaired Student's *t* test, *t*_(10)_ = 10.55). This increase rapidly returned to baseline after stimulation ended.

Similarly, aversive stimulation via an air-puff during NREM sleep induced a significant, transient increase in GLUT^DpMe^ activity, comparable with that elicited by GABA^BNST^ neuronal activation (Fig. 3*E*; No air-puff, *n* = 6, 0.4 ± 0.35%; Air-puff, *n* = 6, 8.58 ± 1.1%, *p* = 0.0002; two-tailed, unpaired Student's *t* test, *t*_(10)_ = 5.588). Due to the brief duration of the air-puff (∼1 s), the activity spike was short-lived. Additionally, auditory stimulation (tone, 2 kHz, 80 dB SPL, 5 s duration) during NREM sleep also induced arousal-associated increases in GLUT^DpMe^ activity, but the response amplitude was smaller (Fig. 3*F*; No sound, *n* = 3, 0.15 ± 0.38%; Sound, *n* = 3, 3.64 ± 0.06%, *p* = 0.0018; Welch's *t* test, *t*_(3.439)_ = 8.710). The peak amplitude of Ca^2+^ transients did not differ significantly between air-puff and optogenetic stimulation (*p* = 0.9992, two-tailed, unpaired Student's *t* test, *t*_(10)_ = 0.09591), whereas the auditory-evoked response was significantly lower than both (vs optogenetic stimuli, *p* = 0.0003, Welch's *t* test, *t*_(6.928)_ = 6.837; vs aversive stimuli, *p* = 0.0106, Welch's *t* test, *t*_(5.905)_ = 3.686).

To compare the effects of these stimuli on sustained arousal, we measured wakefulness after stimulation. Both ChR2-mediated activation of GABA^BNST^ terminals and air-puff stimulation significantly increased wakefulness during the first 3 min poststimulation, with similar levels of arousal in both conditions (Fig. 3*G*; +3 min: EYFP: 15.33 ± 1.26%; ChR2: 90.83 ±2.85%, *p* < 0.0001; Air-puff: 72.5 ± 4.15%, *p* < 0.0001; +6 min: EYFP: 21.5 ± 2.064%; ChR2: 57.83 ± 10.1%, *p* = 0.0252; two-way repeated-measures ANOVA; time, *F*_(1.405,21.07)_ = 72.51, *p* < 0.0001; group, *F*_(2,15)_ = 18.59, *p* < 0.0001; interaction, *F*_(6,45)_ = 11.72, *p* < 0.0001; post hoc comparisons, Dunnett's multiple-comparisons test). These findings were further supported by heatmap analyses (Fig. 3*H*), showing sustained wakefulness in the ChR2 group and a transient increase in the air-puff group.

### DpMe glutamatergic neurons are essential for GABA^BNST^-induced wakefulness

**Figure 4.**
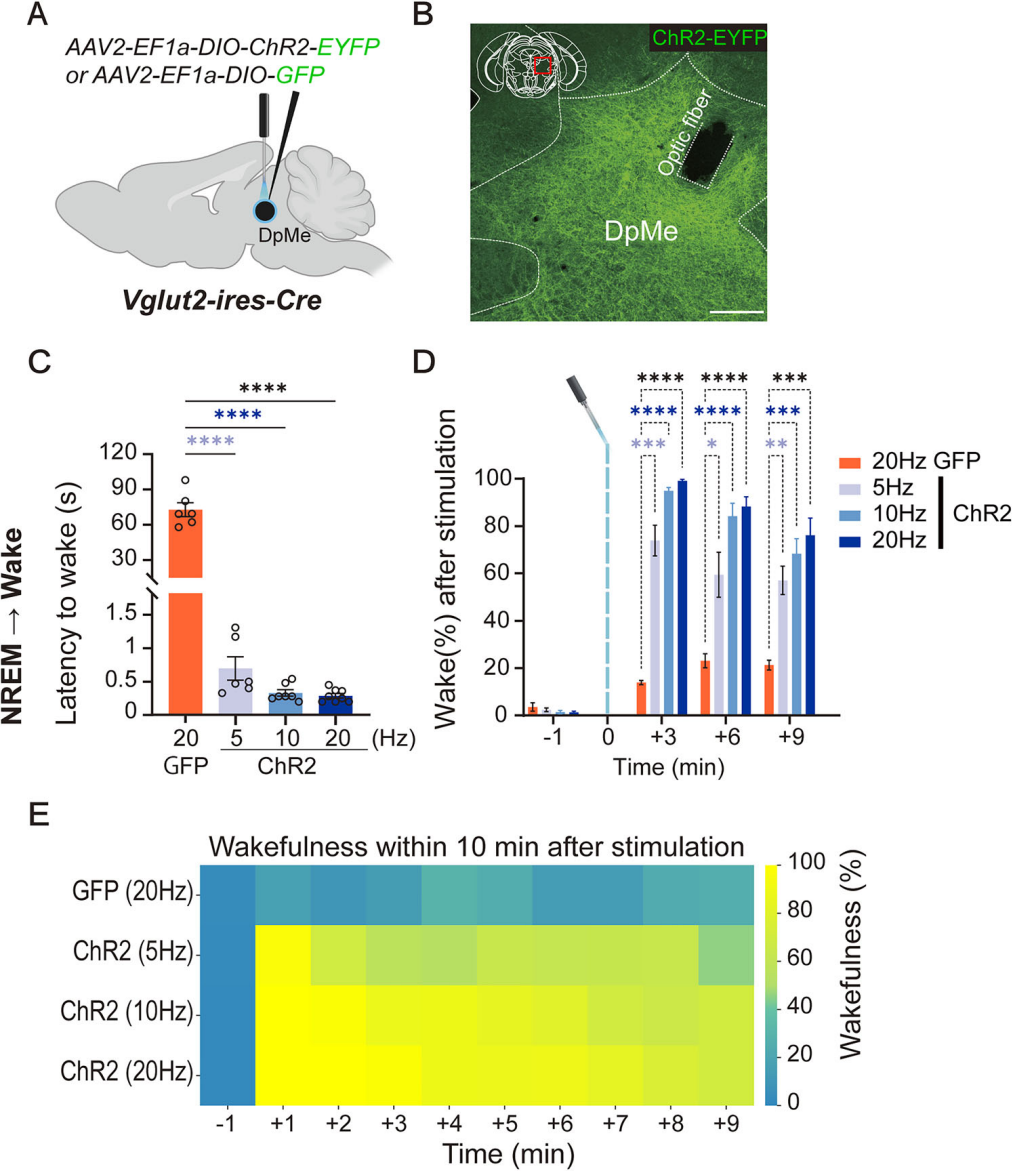
Optogenetic activation of glutamatergic DpMe neurons induces rapid transitions from NREM sleep to wakefulness. ***A***, Schematic of optogenetic experiment in *Vglut2-ires-Cre* mice. *AAV2-EF1a-DIO-ChR2-EYFP* or *AAV2-EF1a-DIO-GFP* was injected into the DpMe to express ChR2 or GFP in glutamatergic neurons. ***B***, Representative image showing ChR2-EYFP expression in DpMe glutamatergic neurons and placement of the optic fiber. Scale bar, 250 µm. ***C***, Quantification of latency from NREM sleep to wakefulness following optogenetic stimulation. ChR2-expressing animals showed significantly reduced latency compared with GFP controls. GFP: *n* = 7; ChR2 (5, 10 and 20 Hz): *n* = 7 per group. Ordinary one-way ANOVA revealed a significant effect of stimulation frequency (*F*_(3,23)_ = 180.1, *p* < 0.0001). Post hoc Dunnett's multiple-comparisons test showed that all ChR2 groups (5, 10, and 20 Hz) exhibited significantly shorter latencies compared with GFP controls (*p* < 0.0001 for all). ****p < 0.0001. ***D***, Wakefulness percentages in 3 min time bins after stimulation. All ChR2 groups exhibited significantly increased wakefulness compared with GFP controls in the first 6 min poststimulation. Two-way repeated-measures ANOVA revealed significant main effects of time (*F*_(2.063,47.44)_ = 290.7, *p* < 0.0001) and group (*F*_(3,23)_ = 48.24, *p* < 0.0001), as well as a significant interaction between time and group (*F*_(9,69)_ = 21.92, *p* < 0.0001). Post hoc Dunnett's multiple-comparisons test showed that at +3 min, all ChR2 groups (5, 10, and 20 Hz) exhibited significantly higher percentages compared with GFP controls (*p* < 0.0001 for 20 and 10 Hz; *p* = 0.0005 for 5 Hz). *p < 0.05, **p < 0.01, ***p < 0.001, ****p < 0.0001. ***E***, Heatmap showing wakefulness percentage over a 10 min period following optogenetic stimulation. Each row corresponds to a stimulation condition.

Having established that activation of GABA^BNST^ neurons increases GLUT^DpMe^ neuronal activity (Fig. 3*D*), we next tested whether direct activation of GLUT^DpMe^ neurons could independently induce wakefulness. To this end, we optogenetically stimulated *Vglut2-*expressing neurons in the DpMe during NREM sleep (Fig. 4*A*,*B*). Compared with GFP controls, ChR2-expressing mice exhibited significantly reduced latencies to wakefulness at all stimulation frequencies (Fig. 4*C*; GFP-20 Hz, *n* = 6, 72.88 ± 5.89 s; ChR2-20 Hz, *n* = 8, 0.29 ± 0.03 s, *p* < 0.0001; ChR2-10 Hz, *n* = 7, 0.33 ± 0.05 s, *p* < 0.0001; ChR2-5 Hz, *n* = 6, 0.70 ± 0.17 s, *p* < 0.0001; one-way ANOVA, *F*_(3,23)_ = 180.1, *p* < 0.0001).

In addition, all ChR2 groups showed significantly elevated wakefulness during the 9 min period following stimulation compared with controls (Fig. 4*D*,*E*; GFP-20 Hz, 13.92 ± 0.91%; ChR2-20 Hz, 99.11 ± 0.61%, *p* < 0.0001; ChR2-10 Hz, 94.86 ± 1.44%, *p* < 0.0001; ChR2-5 Hz, 73.88 ± 6.51%, *p* = 0.0005; two-way repeated-measures ANOVA; time, *F*_(2.063,47.44)_ = 290.7, *p* < 0.0001; group, *F*_(3,23)_ = 48.24, *p* < 0.0001; interaction, *F*_(9,69)_ = 21.92, *p* < 0.0001; post hoc comparisons, Dunnett's multiple-comparisons test). Although wakefulness gradually declined over time, no significant differences were observed among the ChR2 frequency groups, suggesting a saturation effect at relatively low stimulation rates.

**Figure 5.**
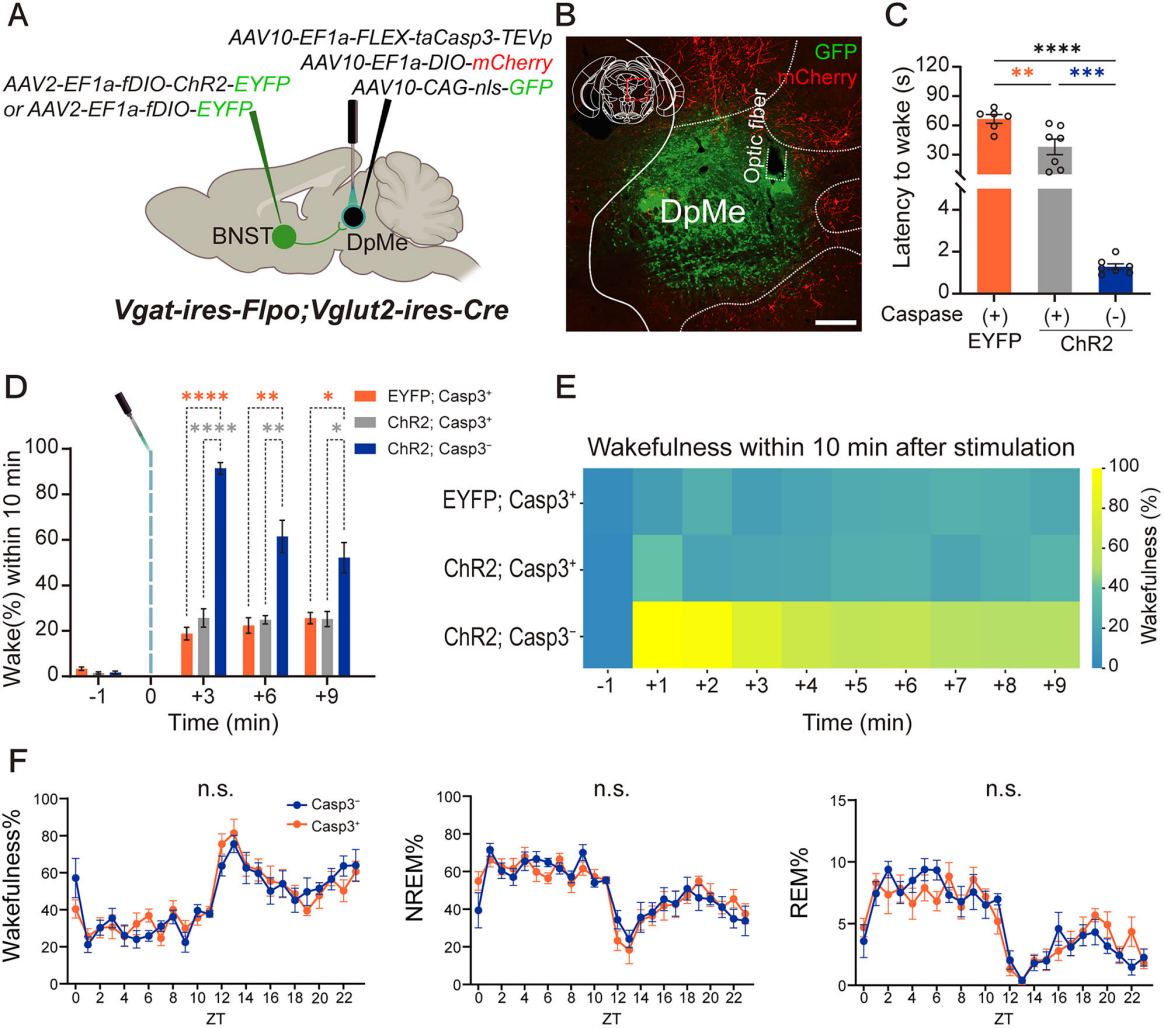
Ablation of glutamatergic DpMe neurons reduces GABA^BNST^ stimulation-induced wakefulness without altering baseline sleep architecture. ***A***, Schematic of triple-virus experimental design in *Vgat-ires-FlpO;Vglut2-ires-Cre* mice. *AAV2-EF1a-fDIO-ChR2-EYFP* or *AAV2-EF1a-fDIO-EYFP* was injected into the BNST, and *AAV10-EF1a-FLEX-taCasp3-TEVp* (with *AAV10-CAG-nls-GFP* and *AAV10-EF1a-DIO-mCherry*) was injected into the DpMe to ablate glutamatergic neurons. ***B***, Representative immunohistochemistry showing BNST-derived axonal projections in the DpMe and viral injection site verification. Punctate, soma-restricted GFP fluorescence marks the injection site in the DpMe, while linear EYFP-positive fibers correspond to BNST axons terminating in the DpMe. The absence of mCherry signal in the DpMe indicates successful caspase-mediated ablation of glutamatergic neurons. Scale bar, 250 µm. ***C***, Quantification of latency from NREM sleep to wakefulness following optogenetic stimulation of GABA^BNST^ neurons. Mice with ChR2 expression but caspase-mediated DpMe ablation exhibited prolonged latencies compared with noncaspase ChR2-expressing controls. Ordinary one-way ANOVA revealed a significant group effect (*F*_(2,17)_ = 36.51, *p* < 0.0001). Post hoc Dunnett's multiple-comparisons test showed that optogenetic stimulation of GABA^BNST^ neurons rapidly induced arousal in (ChR2; Casp3−) mice, whereas ablation of GLUT^DpMe^ neurons delayed awakening (vs ChR2; Casp3+, *p* = 0.0002). Latency in (ChR2; Casp3+) mice was also significantly shorter than in (GFP; Casp3+) controls (*p* = 0.0018). **p < 0.01, ***p < 0.001, ****p < 0.0001. ***D***, Wakefulness percentages across 3 min time bins after stimulation. Noncaspase ChR2 animals showed significantly increased wakefulness compared with both Caspase-ChR2 and Non-ChR2 controls. Two-way repeated-measures ANOVA revealed significant main effects of time (*F*_(2.595,44.11)_ = 118.2, *p* < 0.0001) and group (*F*_(2,17)_ = 52.25, *p* < 0.0001), as well as a significant interaction between time and group (*F*_(6,51)_ = 31.99, *p* < 0.0001). Post hoc Dunnett's multiple-comparisons test showed that wakefulness during the 3 min poststimulation period was markedly reduced in (ChR2; Casp3+) mice compared with (ChR2; Casp3−) mice (*p* < 0.0001) and was statistically indistinguishable from (GFP; Casp3+) controls (*p* = 0.3705). *p < 0.05, **p < 0.01, ****p < 0.0001. ***E***, Heatmap showing the percentage of wakefulness during the 10 min following stimulation. Each row corresponds to a stimulation condition. ***F***, Baseline sleep–wake architecture—measured as the percentage of time spent in NREM, REM, and wakefulness across Zeitgeber time (ZT 0–11, light phase) showed no significant differences, two-way repeated-measures ANOVA.

To determine whether GLUT^DpMe^ neurons act as downstream effectors of GABA^BNST^ neurons, we next performed selective ablation of GLUT^DpMe^ neurons using Cre-dependent Caspase-3 expression in *Vglut2-ires-Cre* mice, followed by optogenetic stimulation of GABA^BNST^ neurons during NREM sleep (Fig. 5*A*,*B*).

In mice with intact GLUT^DpMe^ neurons (ChR2; Casp3−), optogenetic stimulation of GABA^BNST^ neurons induced rapid arousal (Fig. 5*C*; 1.29 ± 0.14 s, *n* = 7). In contrast, mice with ablated GLUT^DpMe^ neurons (ChR2; Casp3+) showed significantly delayed awakening (38.1 ± 7.98 s, *n* = 7, *p* = 0.0002 vs Casp3− group), indicating the necessity of GLUT^DpMe^ neurons in mediating the wake-promoting effects of GABA^BNST^ input. The latency in the ChR2; Casp3+ group remained shorter than that in the GFP; Casp3+ control group (66.61 ± 4.41 s, *n* = 6, *p* = 0.0018 vs control), suggesting partial engagement of other arousal pathways (one-way ANOVA, *F*_(2,17)_ = 36.51, *p* < 0.0001).

Wakefulness during the 3 min poststimulation period was also significantly impaired in the ChR2; Casp3+ group compared with the ChR2; Casp3− group (25.67 ± 4.03% vs 91.41 ± 2.58%, *p* < 0.0001) and was statistically indistinguishable from that of GFP; Casp3+ controls (18.77 ± 2.77%, *p* = 0.3705; Fig. 5*D*,*E*; two-way repeated-measures ANOVA; time, *F*_(2.595,44.11)_ = 118.2, *p* < 0.0001; group, *F*_(2,17)_ = 52.25, *p* < 0.0001; interaction, *F*_(6,51)_ = 31.99, *p* < 0.0001; post hoc comparisons, Dunnett's multiple-comparisons test). These results demonstrate that GLUT^DpMe^ neurons are critical for both initiating and sustaining arousal in response to GABA^BNST^ neuronal activation.

Importantly, gross sleep–wake architecture did not differ significantly between experimental groups (Fig. 5*F*), indicating that ablation of GLUT^DpMe^ neurons does not overtly disrupt spontaneous vigilance states. However, more detailed analysis revealed subtle changes in REM sleep dynamics, including prolonged REM episode duration and increased REM latency, suggesting a possible involvement of these neurons in regulating REM sleep transitions (Table 1).

**Table 1.** Sleep–wake parameters in mice with ablation of DpMe glutamatergic neurons

		WAKE		NREM		REM	
		Casp3+	Casp3−	Casp3+	Casp3−	Casp3+	Casp3−
24 h							
Total time	min	636.3 ± 15.4	627.4 ± 15.9	771.4 ± 16.9	706.3 ± 15.9	71.9 ± 2.1	71.3 ± 1.8
Episode duration	s	151.5 ± 9.9	165.2 ± 10.7	129.3 ± 2.1	109.7 ± 1.5	**60.7 ± 1.7**	50.1 ± 1.3
Number of episodes (R)						**71 ± 2**	85 ± 3
REM latency	s					**185.4 ± 4.9**	134.3 ± 3.4
Inter-REM interval	min					**18.3 ± 0.8**	14.8 ± 0.5
Light period							
Total time	min	214.5 ± 5.4	204.0 ± 7.5	430.6 ± 6.9	428.7 ± 5.8	51.5 ± 2.4	53.2 ± 1.1
Episode duration	s	90.6 ± 7.4	92.5 ± 7.5	138.7 ± 2.8	117.2 ± 2.1	**60.6 ± 2.0**	50.6 ± 1.6
Number of episodes (R)						**51 ± 2**	63 ± 2
REM latency	s					**178.8 ± 5.3**	135.1 ± 4.1
Inter-REM interval	min					**12.4 ± 0.5**	9.7 ± 0.3
Dark period							
Total time	min	421.8 ± 14.0	423.4 ± 17.7	280.8 ± 11.6	277.7 ± 10.7	20.4 ± 1.6	18.2 ± 1.4
Episode duration	s	230.3 ± 20.4	265.7 ± 22.9	117.1 ± 3.1	99.9 ± 2.1	**61.2 ± 2.9**	48.7 ± 2.5
Number of episodes (R)						20 ± 2	22 ± 1
REM latency	s					**202.7 ± 11.1**	132.1 ± 5.8
Inter-REM interval	min					32.6 ± 3	28 ± 1.8

Sleep architecture was assessed in mice with targeted ablation of glutamatergic neurons in the deep mesencephalic nucleus (Casp3+, *n* = 6) and compared with control mice expressing mCherry (Casp3−, *n* = 6). EEG/EMG recordings were performed under baseline conditions, and total time spent in wakefulness (Wake), non-rapid eye movement (NREM) sleep, and rapid eye movement (REM) sleep was quantified over 24 h and across light/dark periods. Data are presented as mean ± SEM. Statistical comparisons between Casp3+ and Casp3− mice were performed using two-tailed, unpaired Student's *t* test. Statistically significant differences are highlighted in bold (24 h: Episode duration, *p* = 0.0008, *t*_(18)_ = 4.017; Number of episodes, *p* = 0.0025, *t*_(18)_ = 3.515; REM latency, *p* = 0.0009, *t*_(18)_ = 3.98; Inter-REM interval, *p* = 0.0014, *t*_(18)_ = 3.756; Light, Episode duration, *p* = 0.0005, *t*_(18)_ = 4.216; Number of episodes, *p* = 0.0043, *t*_(18)_ = 3.261; REM latency, *p* = 0.0007, *t*_(18)_ = 4.093; Inter-REM interval, *p* = 0.0001, *t*_(18)_ = 4.864; Dark, Episode duration, *p* = 0.0044, *t*_(18)_ = 3.251; REM latency, *p* = 0.0357, *t*_(18)_ = 2.27).

### BNST inputs influence DpMe activity via local GABAergic circuits

**Figure 6.**
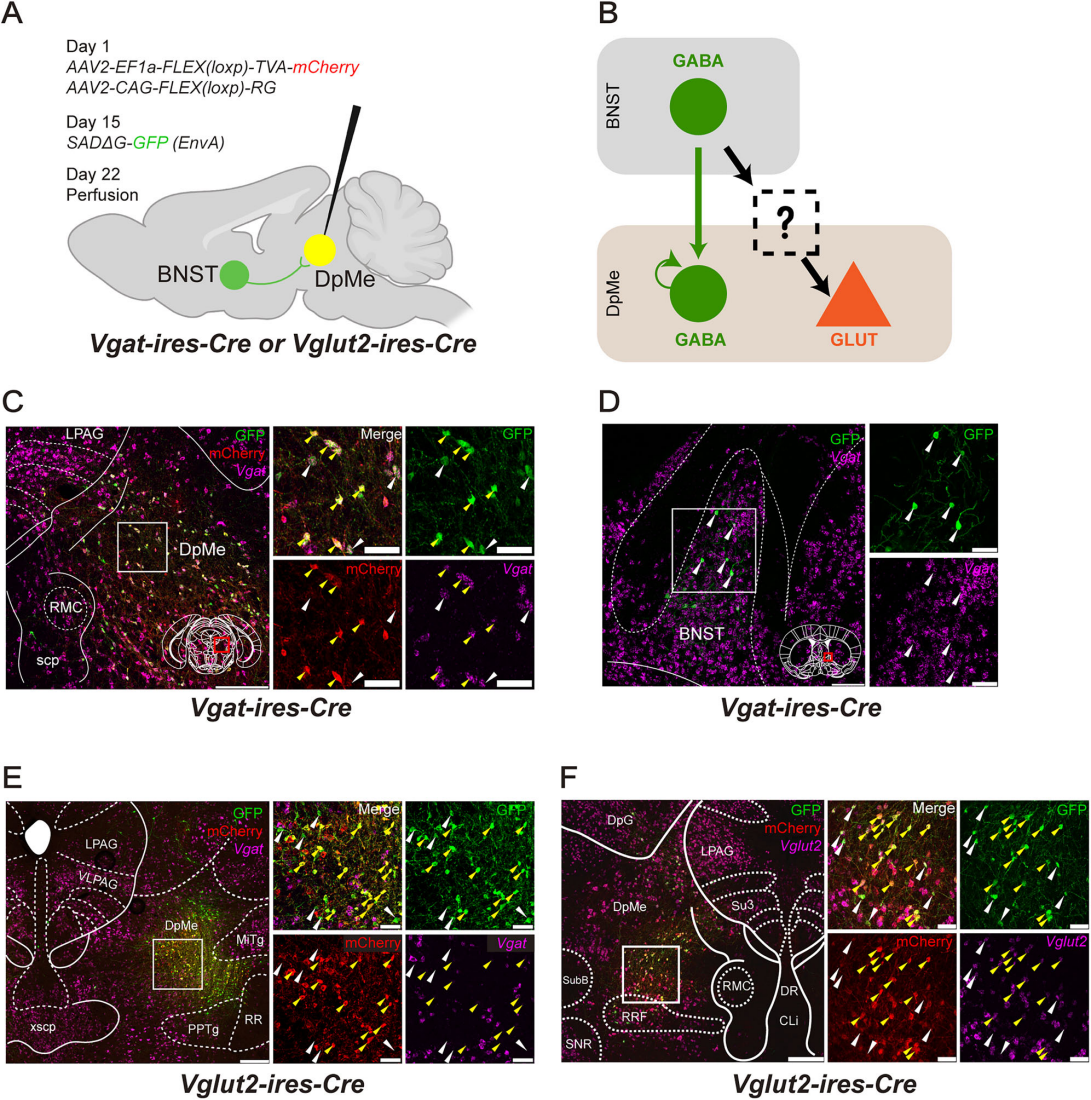
Monosynaptic rabies-based retrograde tracing from DpMe. ***A***, Schematic of monosynaptic retrograde tracing strategy in *Vgat-ires-Cre or Vglut2-ires-Cre* mice. *AAV2-EF1a-FLEX-TVA-mCherry* and *AAV2-CAG-FLEX-RG* were injected into the DpMe, followed by injection of *SADΔG-GFP (EnvA)* at the same site 14 d later. Mice were perfused 7 d after rabies injection (Day 22). Mice brains were analyzed using immunohistochemistry (IHC) and fluorescent in situ hybridization (FISH). Starter neurons were defined as co-expressing mCherry and GFP, whereas input neurons were identified by GFP expression alone. ***B***, Schematic summary of rabies retrograde tracing results. GABA^BNST^ neurons (green, top) project to GABA^DpMe^ neurons (green, bottom), but not directly to GLUT^DpMe^ neurons (orange). Within the DpMe, GABAergic interneurons form recurrent connections onto other GABAergic neurons but show little or no projection to glutamatergic neurons. ***C***, Representative images showing starter and input neurons in the DpMe of *Vgat-ires-Cre* mice. Starter neurons were identified by immunostaining with anti-mCherry and anti-GFP antibodies, while *Vgat*-positive neurons were visualized by FISH. Right, Magnified view of the boxed area in the left image. White arrowheads indicate starter neurons (mCherry^+^/GFP^+^); yellow arrowheads indicate *Vgat*-positive input neurons. Scale bars: left, 250 µm; right, 50 µm. ***D***, Representative images of *Vgat*-positive input neurons in BNST in *Vgat-ires-Cre* mice visualized by IHC and FISH. Right, Magnified image of the boxed area in the left image. White arrowheads indicate *Vgat* and GFP positive input neurons. Scale bars: left, 250 mm; right, 50 mm. ***E***, Representative images showing starter and input neurons in the DpMe of *Vglut2-ires-Cre* mice. Starter neurons were identified by immunostaining with anti-mCherry and anti-GFP antibodies, while *Vgat*-positive neurons were visualized by FISH. Right, Magnified view of the boxed area in the left image. Yellow arrowheads indicate starter neurons (mCherry^+^/GFP^+^); white arrowheads indicate input neurons, all of which were *Vgat*-negative. Scale bars: left, 250 µm; right, 50 µm.

To understand how BNST input shapes the activity of excitatory neurons in the DpMe, we hypothesized that local GABAergic interneurons within the DpMe might serve as intermediaries. To test this possibility, we first mapped the synaptic inputs to GABAergic neurons in the DpMe (GABA^DpMe^ neurons) using monosynaptic retrograde tracing with glycoprotein (G)-deleted, EnvA-pseudotyped rabies virus [SADΔG-GFP(EnvA)] in *Vgat-ires-Cre* mice (Fig. 6*A*). Starter neurons were identified by coexpression of GFP and mCherry (yellow arrowheads, GFP+/mCherry+; Fig. 6*C*). Input neurons, marked by GFP expression alone (white arrowheads, GFP^+^/mCherry^−^/*Vgat*^+^), were observed in both the DpMe and the BNST (Fig. 6*C*,*D*). In situ hybridization confirmed that these input neurons were GABAergic, supporting the presence of direct GABAergic projections from the BNST to GABA^DpMe^ neurons, as well as local GABA→GABA connectivity within the DpMe.

We next conducted a similar tracing experiment in *Vglut2-ires-Cre* mice to identify inputs to glutamatergic neurons in the DpMe (GLUT^DpMe^; Fig. 6*A*). Starter cells (GFP+/mCherry+) and input neurons (GFP+/mCherry−) were again found within the DpMe (Fig. 6*E*), but notably, almost no input neurons were detected in the BNST (Fig. S1). Moreover, the DpMe-localized input neurons lacked *Vgat* expression, indicating they were not GABAergic (Fig. 6*F*).

These findings suggest that GABA^BNST^ neurons do not directly innervate GLUT^DpMe^ neurons. Instead, they likely act via local inhibitory interneurons—GABA^DpMe^ neurons—that in turn regulate the excitability of GLUT^DpMe^ neurons (Fig. 6*B*). This organization supports a disinhibitory mechanism by which BNST inputs relieve inhibition on arousal-promoting GLUT^DpMe^ neurons, thereby facilitating transitions to wakefulness in response to emotionally salient stimuli.

## Discussion

### The role of the GABAergic BNST–DpMe pathway in arousal regulation

We previously demonstrated that optogenetic stimulation of GABA^BNST^ neurons during NREM sleep rapidly induces wakefulness (Kodani et al., 2017). In the present study, we used *Vgat-ires-Cre* mice, instead of *Gad67-Cre* mice, for improved specificity, as GAD67 is also expressed in subsets of glutamatergic neurons (Sharpe et al., 2017). Using this refined model, activation of GABA^BNST^ neurons reliably triggered immediate arousal and increased *c-fos* mRNA expression in the DpMe (Fig. 1). While BNST projections to multiple regions regulate diverse behavioral states, our results highlight the DpMe as a key output mediating emotionally salient arousal from NREM sleep.

Optogenetic stimulation of GABA^BNST^ terminals in the DpMe and direct activation of GLUT^DpMe^ neurons both produced rapid NREM-to-wake transitions (Figs. 2, 4). Fiber photometry recordings confirmed that GABA^BNST^ stimulation transiently increased Ca^2+^ activity in GLUT^DpMe^ neurons. In contrast, stimulation during REM sleep failed to induce arousal, suggesting state-dependent effects: GABA^BNST^ neurons are already active during REM (Li et al., 2024), and REM sleep appears to be stabilized by intrinsic brainstem and forebrain mechanisms that actively resist transitions to wakefulness. These REM-stabilizing processes likely prevent additional BNST input from triggering arousal, even under optogenetic excitation. Although both BNST and DpMe neurons exhibit heightened activity during REM sleep, likely reflecting the well-documented engagement of the extended amygdala in this state, our current findings suggest that the BNST → DpMe pathway specifically promotes arousal from NREM sleep in response to emotionally salient stimuli. The role of these neurons during REM sleep remains to be fully elucidated. In particular, the potential dynamics and functional contributions of DpMe GABAergic neurons during REM were not addressed in this study but represent an important direction for future investigation.

GLUT^DpMe^ neurons were also strongly activated by aversive sensory stimuli (Fig. 3), implicating them in emotionally driven arousal. Their necessity was verified by Caspase-3-mediated ablation, which markedly reduced wakefulness induced by GABA^BNST^ stimulation. Although ablation did not broadly alter baseline sleep architecture (Fig. 5*F*), it modestly prolonged REM duration and latency (Table 1), suggesting a role in REM termination or initiation. The paradox of REM-active neurons contributing to REM termination implies complex state-dependent functions. Overall, these data support an allostatic model in which the BNST → DpMe pathway is recruited under emotionally salient or stressful conditions while subtly modulating REM transitions in baseline states.

The BNST is well known for its role in mediating sustained fear and anxiety (Kim et al., 2013; Goode et al., 2019), and recent studies have shown that GABA^BNST^ neurons are particularly active during wakefulness and REM sleep (Li et al., 2024), aligning with their role in arousal promotion. Our findings extend these observations by identifying the DpMe as a key downstream target of BNST activity. The GABA^BNST^ → GABA^DpMe^ → GLUT^DpMe^ circuit may enable emotionally salient inputs to override sleep pressure and rapidly induce wakefulness. Occasional spontaneous activation of GLUT^DpMe^ neurons preceding natural NREM-to-wake transitions (Fig. 3*C*) suggests that this population may also participate in endogenous arousal generation. Further studies will be necessary to determine the extent to which endogenous activity in this population contributes to natural sleep–wake regulation.

### Circuit architecture of the BNST–DpMe pathway

Using monosynaptic retrograde tracing with rabies virus, we found that GABA^BNST^ neurons form direct synaptic connections with GABAergic neurons in the DpMe, but not with GLUT^DpMe^ neurons (Fig. 6). While our functional data support a model in which GABA^BNST^ neurons promote wakefulness via activation of GLUT^DpMe^ neurons, our anatomical tracing results do not confirm a direct disinhibitory pathway mediated by local GABAergic interneurons in the DpMe. Specifically, monosynaptic rabies tracing from Vglut2-expressing neurons did not reveal strong GABAergic input from within the DpMe. This discrepancy suggests that the circuit architecture may be more complex than a simple disinhibitory loop. One possible explanation is that GABA^BNST^ neurons may indirectly excite GLUT^DpMe^ neurons through polysynaptic circuits involving diverse local interneuron subtypes that are not captured by our current rabies labeling strategy.

Another possibility is that GABAergic transmission itself may exert depolarizing (excitatory) effects under specific physiological conditions, such as altered chloride gradients, which have been observed in subcortical arousal systems (Brown and McKenna, 2015). Future studies combining high-resolution connectomics, cell-type-specific functional mapping, and in situ hybridization for transmitter markers in rabies-labeled cells will be required to fully delineate the local microcircuit by which GABA^BNST^ input regulates DpMe excitability. Nevertheless, our data demonstrate that GABA^BNST^ stimulation enhances GLUT^DpMe^ activity and promotes arousal, supporting the existence of a functional pathway, even if the precise synaptic intermediates remain to be clarified.

Importantly, GABA^BNST^ neurons are known to project not only to the DpMe but also to other arousal-related regions such as the parabrachial nucleus, lateral hypothalamus, and locus ceruleus (Giardino and Pomrenze, 2021). This broad projection pattern likely supports a distributed arousal system and may explain why ablation of GLUT^DpMe^ neurons only partially impaired wakefulness induced by GABA^BNST^ neuron stimulation (Fig. 5*D*,*E*). It is possible that these parallel pathways compensate for the loss of the DpMe branch, particularly under conditions of strong stimulation.

Finally, our findings have potential pharmacological relevance. The involvement of GABAergic circuits in this arousal-promoting mechanism may help explain how sedative agents, such as benzodiazepines that enhance GABA_A_ receptor signaling, suppress arousal, and promote sleep. A deeper understanding of how inhibitory networks gate excitatory output in arousal centers may inform the development of more selective therapies for sleep and anxiety disorders.

### Implications for stress-related sleep disturbances

The BNST appears to serve as a convergence hub for internal (limbic) and external (sensory) inputs that can override sleep-promoting signals and trigger arousal. Our study provides mechanistic insight into how emotionally salient stimuli may induce abrupt transitions from sleep to wakefulness via the BNST–DpMe circuit. Given the known involvement of the BNST in stress and anxiety, this pathway may play a central role in the pathophysiology of stress-related sleep disorders.

Hyperactivity within the BNST has been reported in both animal models and human patients with posttraumatic stress disorder (PTSD) and is associated with persistent hyperarousal and fragmented sleep (Pace-Schott et al., 2015; Shackman et al., 2016). In rodents, elevated BNST activity leads to increased vigilance and impaired sleep, even in the absence of external threats. Notably, corticotropin-releasing factor (CRF)-expressing neurons within the BNST are highly responsive to stress and contribute to prolonged wakefulness (Shackman and Fox, 2016). These findings are echoed in human neuroimaging studies showing that BNST hyperactivation correlates with difficulties in sleep initiation and maintenance among PTSD patients (Silberman and Winder, 2013). Our data suggest that one mechanism by which the BNST disrupts sleep is through disinhibition of GLUT^DpMe^ neurons, enabling emotionally charged signals to promote arousal even during states of strong sleep pressure. Additionally, *prepronociceptin (PNOC)*-expressing GABA^BNST^ neurons have been shown to encode motivational salience and drive rapid arousal responses (Rodriguez-Romaguera et al., 2020), consistent with our proposed model.

Our findings demonstrate that activation of the BNST–DpMe pathway promotes rapid arousal from NREM sleep and that glutamatergic neurons in the DpMe are essential for this effect. It is well established that BNST neurons are recruited under aversive or anxiogenic conditions, as shown in previous studies (Kim et al., 2013). However, the BNST comprises heterogeneous neuronal populations that project to multiple downstream regions involved in diverse functions, including emotion, arousal, and autonomic control. We propose that the BNST → DpMe projection constitutes one such output channel that selectively contributes to arousal regulation, without necessarily mediating emotional expression or behavioral valence.

In this framework, the activation of DpMe glutamatergic neurons by aversive stimuli does not imply that this pathway is intrinsically aversive. Rather, we interpret this as a mechanism by which emotionally salient stimuli, regardless of valence, can trigger rapid transitions to wakefulness. Future studies using behavioral paradigms such as real-time place preference/avoidance may further delineate the affective properties of this circuit.

Together, these results support the idea that the BNST–DpMe pathway functions as a state-switching mechanism, facilitating arousal in response to emotionally salient signals, without necessarily encoding emotional valence itself.

Recent studies have demonstrated that the BNST and the orexin (hypocretin) system are functionally interconnected, suggesting that orexinergic tone may modulate BNST-driven arousal. The BNST sends direct projections to orexin neurons in the lateral hypothalamus while also receiving reciprocal orexinergic inputs that influence stress and motivated behaviors (Sakurai, 2014; Giardino et al., 2018). Given that orexin neurons are key mediators of wakefulness and emotional arousal (Sakurai, 2007), it is plausible that the BNST–DpMe pathway identified in the present study operates in concert with the orexin system. Future experiments combining BNST–DpMe manipulation with monitoring or modulation of orexin neuronal activity will be valuable for clarifying how these systems cooperate to regulate wakefulness under emotional contexts.

Together, these findings point to the BNST–DpMe circuit as a potential therapeutic target for alleviating hyperarousal and sleep fragmentation associated with stress-related psychiatric conditions. Interventions aimed at modulating this pathway may help restore normal sleep–wake transitions in individuals affected by trauma, anxiety, or other forms of emotional dysregulation.
